# 

**Published:** 1916-10-14

**Authors:** 


### CONTENTS.

PAGE

**Medical Science and Ethics in War** ... ... 19

**Suggested Isolation of Wounded Canadians** ... 20

**Hospital and Institutional News** ... ... ... 21

The Diamond Jubilee of the Great Northern Central Hospital—The Memory of Edith Cavell—Proposal to Change the Name of a Hospital—The New Leeds Hospital Block—Patriotism at Bradford—A Military Orthopædic Hospital at Cardiff—An Unfortunate Controversy at Leeds—Hospital's Half-Mile of Pennies—Birmingham Hospital Saturday Fund Distribution—The Birmingham Orthopædic Hospital—Extension of the Military Hospital at Brighouse—First Aid at the Royal College of Surgeons—A Serious Allegation against Nursing Homes—New Work at Worcester Infirmary—The Fate of the Military Mental Patient—The Melbourne Hospital and the State—The Lesson of Consumption Statistics—Wages and Disease: An Economic Paradox—Butter *versus* Margarine in Hospitals—Tobacco and the Soldier—Anti-Venereal Crusade in Derbyshire—Death of an Ex-Hospital Surgeon—Death of a Gallant Medical Officer—The Friendly Society Dispute in New Zealand—The Medical Society of London—Panel Doctors and Consultants—This Week's Drug Market.

**The Health of London...** ... ... ... ... 27

Report of the County Medical Officer of Health and School Medical Officer (L.C.C.) for 1915.

**Epidemics Resulting from Wars** ... ... ... 28

Typhus and Other Pestilences.

**Teaching at the Royal Sanitary Institute** ... 30

PAGE

**Life and Death: Some Human Lessons in War-Time** ... ... ... ... ... ... ... 29

IV.—Riches and Wealth: Their Limitations, Use, and Impotence.

**The Institutional Library** ... ... ... ... 31

When to Advise Operation in General Practice—Gray's Anatomy, Descriptive and Applied—The Treatment of Diseases of the Skin—Mental Nursing—Uncensored Letters from the Dardanelles—Our Baby—First Principles in Therapeutics.

**The Matrons' and Sisters' Department** ... 33

The College of Nursing, Limited: Constitution of the Scottish Board.

Round the Hospitals.

The Registration Bill's Prospects—Time for Action—Commandant and Matron—The Training-School at Rheims.

**The Editor's Letter-Box** ... ... ... ... 35

Life and Death: Some Human Lessons in War-time.

**British Hospital and Ambulance Work in France** ... ... ... ... ... ... ... 36

Some Impressions of a Visit.

**Matrons' and Sisters' Appointments** ... ... vi

**The Institutional Worker** (Suppl.) 1

Easy Household Tests for Food Adulteration.

Question for October.

Enquiries and Answers.

The Sick and in Need.

Practical Matters.

